# Noninvasive measurements of hemodynamic, autonomic and endothelial function as predictors of mortality in sepsis: A prospective cohort study

**DOI:** 10.1371/journal.pone.0213239

**Published:** 2019-03-11

**Authors:** Jose Carlos Bonjorno Junior, Flávia Rossi Caruso, Renata Gonçalves Mendes, Tamara Rodrigues da Silva, Thaís Marina Pires de Campos Biazon, Francini Rangel, Shane A. Phillips, Ross Arena, Audrey Borghi-Silva

**Affiliations:** 1 Bioengineering Interunities, USP, Campus São Carlos, Sao Carlos, SP, Brazil; 2 Department of Medicine—Federal University of Sao Carlos, Sao Carlos, Brazil; 3 Cardiopulmonary Physical Therapy Laboratory, Federal University of Sao Carlos, Sao Carlos, Brazil; 4 Integrative Physiology Laboratory, College of Applied Health Sciences, University of Illinois Chicago, Chicago, IL, United States of America; Soroka University Medical Center, ISRAEL

## Abstract

**Background and aim:**

Sepsis is associated with marked alterations in hemodynamic responses, autonomic dysfunction and impaired vascular function. However, to our knowledge, analysis of noninvasive markers to identify greater risk of death has not yet been investigated. Thus, our aim was to explore the prognostic utility of cardiac output (CO), stroke volume (SV), indices of vagal modulation (RMSSD and SD1), total heart rate variability (HRV) indices and FMD of brachial artery (%FMD), all measured noninvasively, in the first 24 hours of the diagnosis of sepsis.

**Methods:**

60 patients were recruited at ICU between 2015 and 2017 and followed by 28 days. CO, SV, RR intervals were measurement. Doppler ultrasound was used to assess brachial artery FMD and the hyperemic response were obtained (%FMD). Patients were divided by survivors (SG) and nonsurvivors groups (NSG).

**Results:**

A total of 60 patients were analysed (SG = 21 and NSG = 39). Survivors were younger (41±15 years vs. 55±11 years) and used less vasoactive drugs. As expected, APACHE and SOFA scores were lower in NSG compared to SG. In addition, higher SD1, triangular index, % FMD, velocity baseline and hyperemia flow velocity as well as lower HR values were observed in the SG, compared to NSG (P<0.05). Interestingly, RMSSD and SD1 indices were independent predictors of %FMD, ΔFMD and FMD_peak_. RMSSD threshold of 10.8ms and %FMD threshold of -1 were optimal at discriminatomg survivors and nonsurvivors.

**Conclusion:**

Noninvasive measurements of autonomic and endotelial function may be important markers of sepsis mortality, which can be easily obtained in the early stages of sepsis at the bedside.

## Introduction

Sepsis has been conceptualized by the presence of life-threatening organic dysfunction, secondary to a dysregulated response to infection [1]; sepsis is associated high mortality in Brazil and other countries around the world [2,3]. Recent study reported 40 to 80% mortality rates with septic shock [4].

Sepsis produces specific alterations in global cardiovascular dynamics (macrocirculation) and microcirculatory blood flow [5] as well as autonomic nervous system control. Much attention has been given to the severity of macro and microcirculatory failure, since they are independent variables and can independently induce organ dysfunction [6]. Micro and macrovascular alterations are a hallmark of sepsis and play a crucial role in its pathophysiology [7]. In addition, early identification of sepsis is one of the most important factors in improving clinical outcomes [8] (i.e., recovery and prognosis).

In this context, impedance cardiography is a noninvasive method of cardiac output monitoring that may be a useful monitor at the bedside for patients in early stages of sepsis in order to assess the dynamics of macrocirculation. A recent study showed that early noninvasive measurement of cardiac index in critically ill severe sepsis and septic shock patients [8] may indicate the severity of disease. In addition, autonomic nervous control noninvasively measured by hear rate variability (HRV) has shown promise as a measure to investigate the balance of parassympathetic and sympathetic modulation during severe inflamation [9]. A recent study observed that several HRV parameters are reduced in nonsurviving septic patients [9].

Ultrasound analysis of brachial artery reactivity in response to ischemia stimulus provides an important estimate of microvascular and endothelial function/dysfunction [10]. Post-cuff occlusion flow-mediated dilation (FMD) is a noninvasive method that have been considered as an indicator of nitric oxide (NO) bioavailability [11]. Recent evidence demonstrates FMD is reduced in septic patients and may be a marker of end organ damage and mortality. Therefore, the aim of the current study was to analyse macrocirculation by cardioimpedance, microcirculation responses by FMD and autonomic nervous control by HRV indices within 24 hours of a sepsis diagnosis. The hypothesis of the present study is that noninvasive tools of macro and microcirculation as well as indices of HRV can identify important differences between survivors and non-survivors.

### Study design, population and protocol overview

This was a prospective study consisting of patients with sepsis. This manuscript followed the recommendations of the STROBE Statement [12].

Sixty males and females, aged between 18 and 70 years were admitted to the intensive care unit (ICU) of the Santa Casa de Misericórdia Hospital of Sao Carlos from August 2015 to September 2017, with diagnosis of sepsis, according to American College of Chest Physicians/Society of Critical Care Medicine ACCP/SCCM [13]. For analysis puroposes, patients were divided into a survivor (SG) and non-survivor group (NSG). In addition, all patients and/or family members signed n informed consent and in accordance with the Ethical Guidelines of the Helsinki Declaration, this study was carried out with the approval of the Federal University of Sao Carlos Ethics Committee (process n° 1.565.648). The present study was registered at ensaiosclinicos.gov.br/rg/ (RBR-7szm34).

The main inclusion criteria were age 18 to 70 years admitted to the ICU with a sepsis diagnosis. Sepsis was defined as an inflammatory response with suspected infection, based on documented medical treatment and/or laboratory results (such as pneumonia, chest radiografy and abscess formation) [14]. In addition, the systemic inflammatory manifestations were based on the following variables: 1) axillary temperature >38°C or hypothermia (axillary temperature <36°C); 2) tachycardia, (heart rate>90 beats per minute); 3) tachypnea (respiratory breathing > 20) or need for mechanical ventilation; 4) leukocytosis (>12,000 cells/mm^3^) or leukopeny (4000 cells/mm^3^); or 5) a ratio greater than 10 polymorphonuclear cells. Severe sepsis was defined as clinical signs associated with organic alteration, change in perfusion or hypotension. Patients were scored according to the Acute Physiology and Chronic Health Evaluation (APACHE II) score. Patients with ventilatory support by invasive or noninvasive mechanical ventilation, as well as patients in spontaneous breathing (ambient air) with oxygen implantation were included. In addition, the SOFA score (Sepsis-related organ failure assessment) was used to assess organ and system failure in critically ill patients. Exclusion criteria consisted of patients who had one or more of the following conditions: 1) under 18 or above 70 years of age; 2) persistent cardiac arrhythmias such as atrial fibrillation, pre-existing atrioventricular block or ventricular tachycardia; 3) cardiogenic shock; 4) pregnancy; 5) cardiac pacemaker; or 6) cancer diagnosis.

### Pre experimental procedures

Initialy, patients admitted to the ICU were monitored and assisted through a multiparameter monitor, including an electrocardiogram (ECG), oximetry and automatic blood pressure and thermometer (Dixtal, DX 2021, Amazonas-Brazil). If the patients needed ventilatory support, the Carmel Takaoka Pulmonary Servo Ventilator, or oxygen support supplementation was administered by means of a high oxygen concentration reservoir mask or through a nasal oxygen catheter. Oxygen supply was required to maintain peripheral oxygen saturation (SpO_2_) ≥ 95%. Patients were evaluated in the supine resting conditionand all procedures were performed in an air-conditioned environment with a temperature between 22 and 24°C and adequate relative humidity (40 to 60%). In addition, tests such as complete blood testing, blood glucose, creatinine, hemoglobin and leukocytes, C-reactive protein, arterial and venous blood gases, and serum lactate levels (according to the APACHE II index) were performed at ICU admission. In this sense, demographic information, clinical data, laboratory tests, admission diagnoses, primary site of infection, length of ICU stay, and length of hospital stay were recorded. Patients who were discharged from the hospital in less than 28 days or who remained alive for 28 days were considered "alive" or "survivors" in the present study. Additional variables needed to calculate the MEDS score (Mortality in Emergency Department Sepsis), as an indicator for predicting mortality, were obtained based on the worst clinical situation during the hospital stay for each patient.

Up to 24 hours after the diagnosis and fulfilling all inclusion criteria, the patients were submitted to the experimental protocol composed by noninvasive hemodynamic analysis, R-R intervals capture and followed by flow-mediated vasodilation (FMD) analysis. During the data collection, patients had controlled temperature (<37.5°C) and stable blood pressure (> 100 and <150 systolic blood pressure and > 60 and <100 for diastolic blood pressure). All medications were anoted. Settings for mechanical ventilatory assistance, if administered, was maintained throughout data collection. The tidal volume, inspiratory time, expiratory time, total time, respiratory rate (RR) and final positive expiratory pressure and inspiratory oxygen fraction were collected. If the patient was in spontaneous breathing, respiratory rate was obtained as well as the type (mask or cateter) and amount of oxygen support used. The patients were placed in the supine position, maintained for 10 min. Conscious patients were instructed not to perform movements and not to talk during the evaluation. In addition, patients were monitored by 12-lead ECG, R-R intervals collected by a heart rate monitor and cardioimpedance monitorization; data collection continued for for 10 minutes in the supine position. After this basal data collection, measurement of FMD through the brachial artery in the supine position was performed.

### Experimental procedures

Central hemodynamics: Measures were obtained noninvasively beat-to-beat using an impedance cardiography device (PhysioFlow PF-05, Manatec Biomedical, France). The principle of PhysioFlow is based on the assumption that variations in impedance (ΔZ) occur when an alternating current of high frequency (75 kHz) and low magnitude (1.8 mA) passes through the thorax during cardiac ejection, thus presenting a specific waveform, from which stroke volume can be calculated [15]. Before data collection, the system was calibrated while taking age, body mass and blood pressure values into consideration. The verification of the correct signal quality was performed by visualising the ECG tracing and its first derivative (dECG/dt) and the impedance waveform (DZ) with its first derivative (dZ/dt), according to recommendations [16]. Measurements of cardiac output (CO = l/min) and stroke volume (SV = l), heart rate (HR, beats) and cardiac index (CI) were obtained.

ECG monitorization and acquisition of R-R interval (RRi): RRi was registered using the Polar system (Polar S810i, Kempele, OL, Finlândia), accompanied by the quality of electrocardiographic signals by means of a single-channel cardiac monitor. HR and RRi were collected for 10min in the supine position. An elastic belt (Polar T31 transmitter, Polar Electro, Kempele, Finland) was attached to the thorax of the patient at the level of the lower third of the sternum. The belt contains a stable case with HR electrodes, an electronic processing unit and an electromagnetic field transmitter. HR signals were continuously transmitted to the Polar Advantage receiver unit via an electromagnetic field. All data were transferred to a computer using Polar Pro-Trainer software. HRV índices were analyzed and calculated using Kubius HRV software (MATLAB, version 2.1, Kuopio, Finland) [17]. The total period of RRi collection was scrutinized and, within a 10-minute period, the most stable noise-independent segment (ie, without missing data and/or noise events) containing 256 data points was selected for analysis. Time and frequency domains as well as nonlinear indices were obtained. The mean RRi, the SD of the normal RR intervals (SDNN) and the square root of the mean squared differences of successive RR intervals (RMSSD) were obtained for time domain linear analysis. Low (LF) and high frequency (HF in normalized units (nu) were calculated. In addition, the LF/HF ratio was calculated to verify the sympathovagal balance [18]. Nonlinear HRV indices was performed from SD1 (standard deviation measuring the dispersion of points in the plot perpendicular to the line-of-identity), SD2 (standard deviation measuring the dispersion of points along the line-of-identity). SD1 represents parasympathetic modulation, while SD2 reflects total variability.

Brachial artery flow-mediated dilation (FMD): The peripheral vessel ultrasound evaluation was performed with a high-resolution ultrasound system (M-Turbo, Sonosite, Bothell, WA, USA) using techniques previously described [19]. Ultrasound imaging of the brachial artery was performed after hemodynamic and RRi data collection. Measurements were obtained with the arm abducted approximately 80^o^ from the body and the forearm supinated. The ultrasound probe (10MHz) was positioned with a 60º insonation angle in a longitudinal plane at a site 1–3 cm proximal to the antecubital fossa, in order to visualize the anterior and posterior lumen-intima interfaces to measure diameter and central flow velocity (pulsed Doppler). After baseline (BSL) images were recorded, a blood pressure cuff, positioned on the forearm, was inflated to 200mmHg for 5 min. To assess FMD, images were acquired continuously for 3 minutes after cuff deflation, during a reactive hyperemia (RH) period. Flow velocity was recorded at BSL and just after cuff release where maximal velocity was observed. All images were digitally recorded for posterior analysis using the software Vascular Research Tools (Medical Imaging Applications, Coralville, IA, USA). Relative FMD was calculated using the largest mean brachial artery diameter at BSL compared with the largest mean values obtained after release of forearm occlusion [%FMD = (RH diameter mm–BSL diameter mm/BSL diameter mm) x 100]. Absolute FMD was calculated as the following: ΔFMD = RH diameter (mm)–BSL diameter (mm). FMD% response to exercise was calculated as following: FMD response = %FMD 15min–%FMD rest [11].

## Data analysis strategy

The sample size calculation was done using Gpower software (3.1.9.2); to achieve a statistical power of 80% (β = 0.20), with α = 0.05, it was calculated that a minimum of 58 subjects were needed, 29 in each group^10^. The null hypothesis tested was that there would be no difference in FMD and the linear and non-linear indices of HRV between survivors and non-survivors. According to the nature of the distribution of the variables, the measures of central tendency and dispersion were means and SDs (parametric). Student's t test was used for anthropometric and clinical (laboratory) variables. Fischer's exact test for sepsis origin and Chi-square test for drug use were applied. Correlation analysis was also performed with the Pearson correlation coefficients; r-values were interpreted as: 1) 0.0–0.19: no correlation; 2) r = 0.20–0.39: weak; 3) r = 0.4–0.69 moderate; 4) r = 0.70–089: strong; and 5) r = 0.9–1.00: very strong. In addition, single linear regression for prediction analyzes were applied. R^2^ with p<0.05 were considered, provided that all prerequisites for prediction models were reached (N = 20, the values of the variables were independent, linear relationship between variables, analysis of residues by Durbin Watson lower than 2.5, limit of outliers below 3DP, and homoscedasticity of the residues). Multiple linear regressions were applied to the data collected of all patients. P<0.05 was considered statistically significant Kaplan–Meier analysis of event-free survival during follow-up was performed on the basis of cut-off values for RMSSD and FMD and survival were studied with Cox regression. A p-value <0.05 was considered statistically significant for all tests. Analyzes and graphs were performed using Statistical Package for Social Sciences (SPSS), version 19.0 (SPSS Inc, Chicago, IL) and Medcalc statistical software (version 11.6.1; Belgium).

## Results

**Fig 1:**
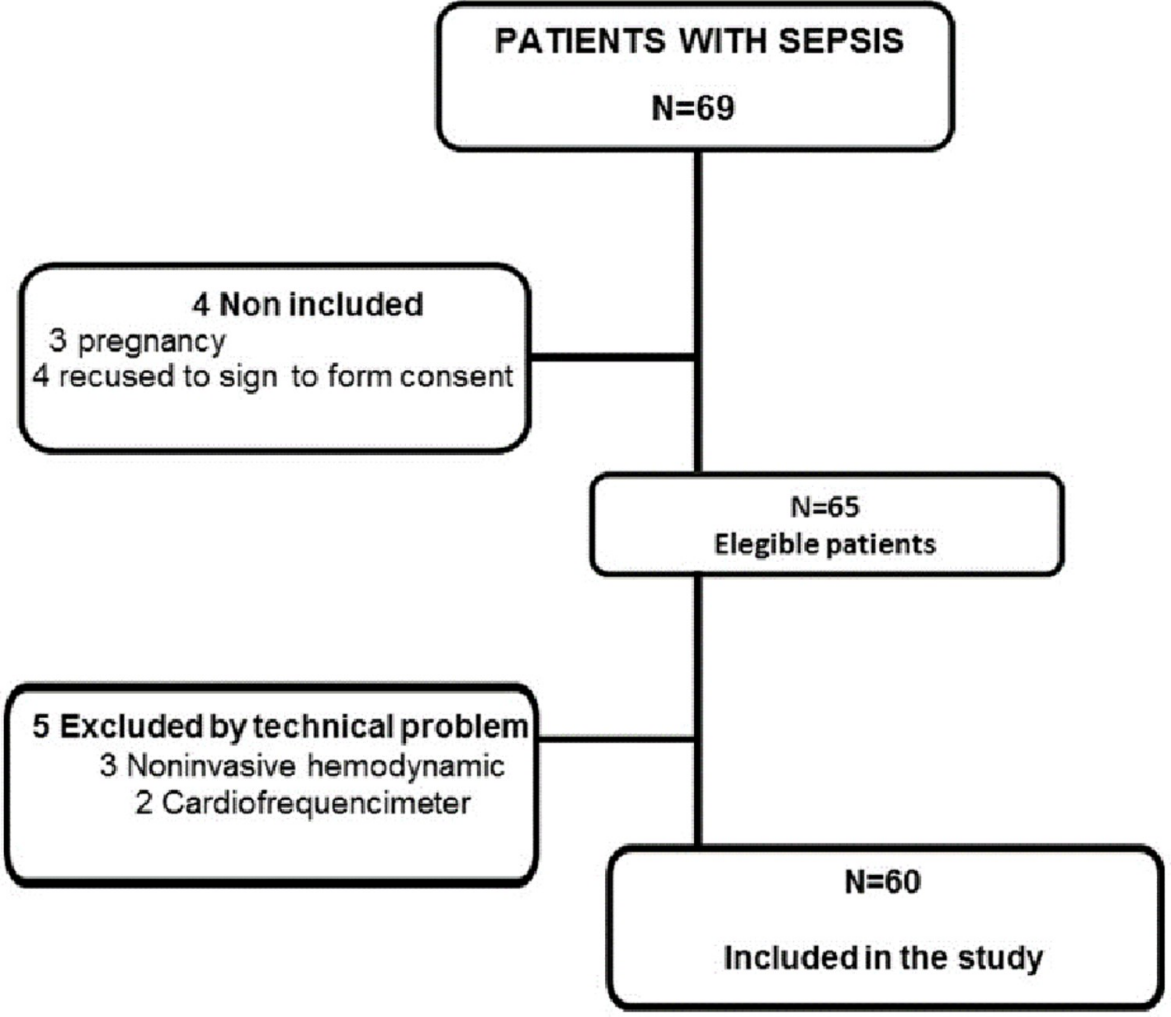
Flowchart of the study.

Sixty-nine patients were initially screened; after the initial evaluation 4 patients were excluded (3 pregnancy and 1 refused to sign the informed consent). Thus 65 were recruited and agreed to participate in the present study. After the evaluation, 5 patients were excluded because of technical problems with data analysis as illustrated in Fig 1.

Anthropometric and clinical characteristics for the 60 volunteers who completed the study are summarized in Table 1. The baseline characteristics of the cohort of septic patients are presented in Table 1, stratified according to 28-day all-cause mortality. As observed, 29 (65%) out of the 60 patients died during the 28 day follow-up. There were no significant differences in baseline characteristics. Regarding the origin of sepsis, the majority of patients (n = 20) in the NSG presented with a respiratory origin when compared to the SG. Regarding the origin of intra-abdominal sepsis, 16 patients of intra-abdominal origin with in the NSG compared to 11 in the SG (p<0.05). In addition, considering clinical variables, the APACHE and SOFA scores, was significantly higher in the NSG when compared to the SG. The same behavior was apparent for APACHE mortality (p<0.05). Regarding medications, the NSG presented with higher administration of noradrenaline and sedatives when compared to the SG as shown in Table 1 (p<0.05). For clinical parameters the SG presented higher venous oxygen content in contrast to the NSG (P = 0.001).

**Table 1:** Baseline characteristics and clinical variables of the patients.

*Parameters*	*Survivors* *N = 21*	*Nonsurvivors N = 39*	*P values*
Age (years)	41.2 ± 14.9	55.2 ± 11.1*	0.007
Height (m)	1.69 ± 0.1	1.65 ± 0.1	0.2
Body mass (Kg)	71.4 ± 13.3	73.1 ± 14.2	0.6
Body Mass Index (Kg/m^2^)	24.7 ± 3.9	26.5 ± 3.9	0.1
Male (n)	15	20	0.5
Female (n)	6	19*	0.02
***Sepsis Origens***			
Respiratory tract (n)	10	20*	0.01
Intra-Abdominal (n)	11	16	1
Others (n)	-	3	
***Clinical variables***			
Mechanical ventilation (n)	21	39	0.05
APACHE score (0–100)	21.9 ± 10.3	28.7 ± 6.1*	0.03
APACHE Mortality (%)	37.2 ± 30.2	61.6 ± 21.5*	0.001
SOFA score (0–24)	6.5 ± 4	10.3 ± 3.1*	0.001
Time Intensive Care Unit	9.6 ± 6.8	12.3 ± 9.6	0.23
Temperature C°	36.7 ± 0.8	37.1 ± 0.9	0.1
Heart rate (bpm)	103.2 ± 23.5	110.2 ± 23.7	0.1
C-reactive protein (mg/L)	19 ± 11.1	19.4 ± 9.7	0.7
Lactate (mmol/L)	2.1 ± 1	2.7 ± 1.7	0.2
pH	7.3 ± 0.07	7.3 ± 0.1	0.1
pO_2_	103.4 ± 22.6	102 ± 34.3	0.8
pCO_2_	38.6 ± 17.2	39.4 ± 13	0.7
pO_2_/FiO_2_ ratio	281.4 ± 110	274.9 ± 105.1	0.8
FiO_2_ (%)	45.4 ± 18.3	40.6 ± 14.2	0.3
Glucose (mg/dL)	126.5 ± 52.3	157.9 ± 77.4	0.1
Antibiotic (n)	21	39	1
Noradrenaline (n)	10	28*	0.01
Dobutamine (n)	3	10	0.08
Sedative (n)	11	41*	0.0004
Hemoglobin (g/dL)	10.1 ± 1.9	9.4 ± 1.7	0.1
Platelet /mm^3^	245.1 ± 152	257.4 ± 138.3	0.3
Leukocytes /mm^3^	30.6 ± 50.1	14.8 ± 0.3	0.1
Hematocrit (%)	31 ± 5.7	18 ± 10.3	0.2
GOT (U/L)	48 ± 15.6	79.6 ± 83.5	1
GPT (U/L)	39.8 ± 21.6	72.3 ± 88.4	0.6
Creatinine mg/dl	2.8 ± 4.2	2.4 ± 2.1	0.1
Gama GT (U/L)	138.5 ± 59	85.5 ± 73.8	0.2
Amylase (U/L)	34 ± 16.9	51.8 ±19.5	0.1
Arterial oxygen content (vol %)	12.9 ± 1.9	12.1 ± 2.4	0.2
Venous oxygen content (vol %)	11.5 ± 2.8	9.4 ± 2.7*	0.001
Venous oxygen saturation (%)	77.1 ± 7	74.1 ± 11.8	0.3

Data in mean ± SD. N = number of patients; Apache = prognostic scoring system for the evaluation of critically patients; SOFA = Sequential Organ Failure Assessment Score; pH = potential of hydrogen; pO_2_ = O_2_ partial pressure; pCO_2_ = CO_2_ partial pressure; FiO_2_ = fraction of inspired oxygen; GOT = glutamic oxaloacetic transaminase; GPT = glutamic—pyruvic transaminase; Gama GT = Gama glutamyl transferase. Student t test for anthropometric and clinical variables, Fischer's exact test for sepsis origins and Qui Square test for medications

* p<0.05.

**Fig 2:**
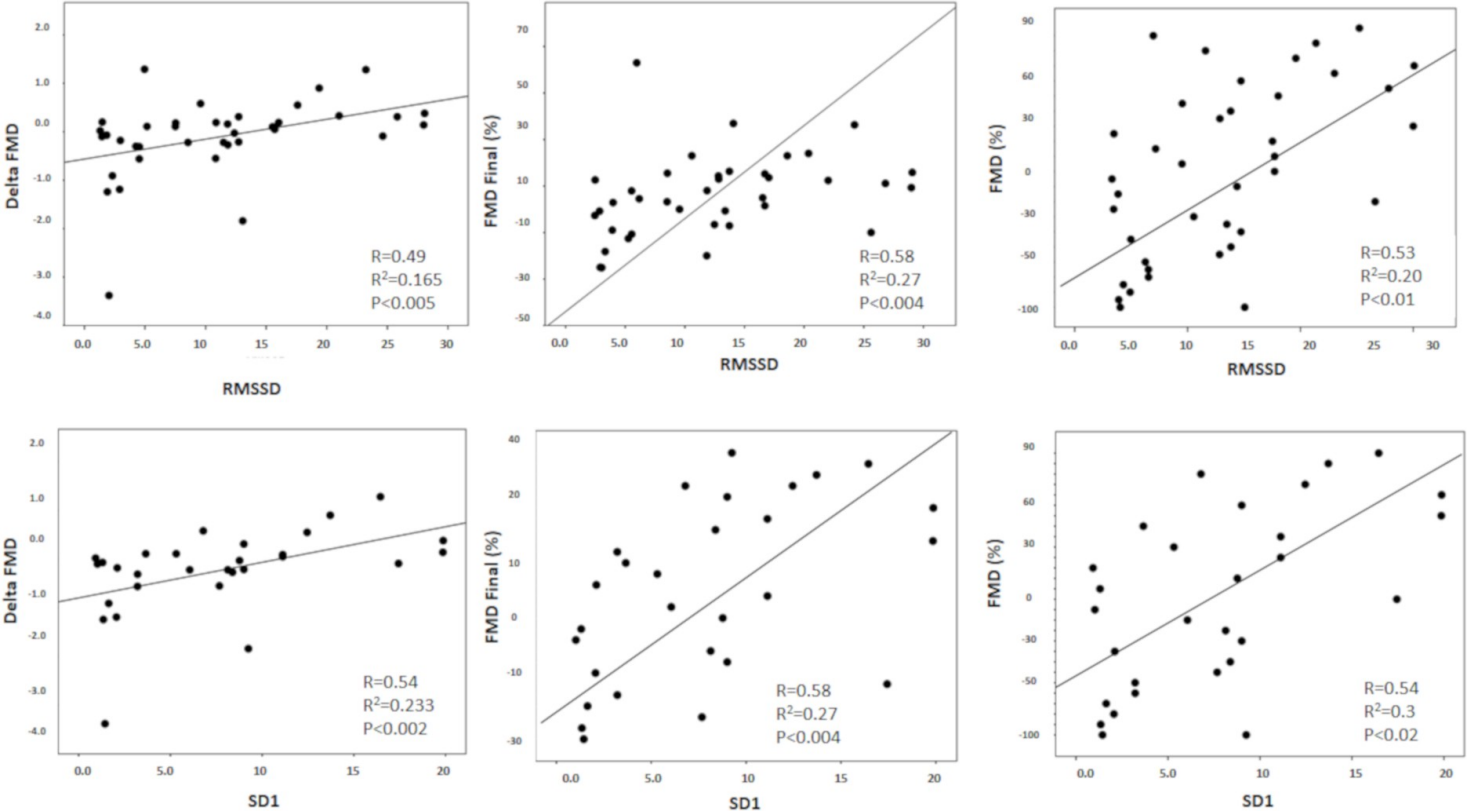
Moderate and positive Pearson correlation and linear regression analysis between vagal autonomic modulation (RMSSD and SD1) and FMD índices (%FMD, FMDpeak and ΔFMD) were found, p<0.05.

Hemodynamic, autonomic and FMD measures of each group are listed in Table 2. Regarding the noninvasive hemodynamic data, we observed that HR presented with significantly higher values in the NSG compared to the SG (p<0.05). In addition, signficantly lower values were observed in the NSG when contrated to the SG for HRV indices (HR mean, RR Tria Index, SD1, P<0.05). FMD data from the brachial artery in patients with sepsis showed that the NSG presented with reduced values of % FMD, velocity baseline and hyperemia flow velocity when compared to the SG (p<0.05). In the multiple linear regression of Table 3, a significant association were identified between the Age and Hypertension variables in patients with sepsis with R^2^ value of 0.72. Thus, it was possible to demonstrate that Age, Hypertension, Diabetes Mellitus and FMD were significant predictors of mortality. Fig 2 illustrates the correlations and univariate regression of vagal modulation indices (RMSSD and SD1) to predict FMD parameters (FMD%, FMDpeak and ΔFMD). We observed moderate correlations between RMSSD and SD1 indices with FMD variables (% FMD, peak FMD and FMD delta); HRV was an independent predictor of FMD (P<0.05).

**Table 2:** Hemodynamic, autonomic and endothelium function data of patients.

	*Survivors* *N = 21*	*Nonsurvivors N = 39*	*P values*
***Hemodynamic variables***			
HR (bpm)	97 ± 13	120 ± 19*	0.005
CO (L/min)	6.8 ± 1,9	6.5± 2.7	0.1
SV (mL)	70 ± 17	56 ± 20	0.4
CI	3.9 ± 0.9	3.5 ± 1.5	0.5
SVR (dynes-sec/cm^5^)	1235 ± 289	1135 ± 355	0.81
***HRV indices***			
***Time Domain***			
RMSSD	14.6 ± 7,6	7.5 ± 4.7	1
Mean HR	84 ± 15	105 ± 27*	0.02
SDNN	21.7 ± 15.1	13.3 ± 11.1	0.08
Triangular index (RRtri)	4.8 ± 2.7	3.5 ± 1.7*	0.008
***Frequency domain indices***			
LF (un)	60.3 ± 25.8	53 ± 19	0.3
HF (un)	39.6 ± 25.7	46 ± 19	0.3
LF/HF	3.0 ± 3.4	1.7 ± 1.6	0.2
***Non Linear Indices***			
SD1	9.5 ± 5.7	5.1 ± 2.9*	0.02
SD2	22.4 ± 21.1	13.8 ± 12.3	0.2
Shannon Entropy	3.1 ± 0.6	3.2 ± 0.7	0.9
SampEn	3.2 ± 6.2	2.3 ± 5.2	0.7
ApEn	8.2 ± 23.4	5.1 ± 20.1	0.7
DFA α1	0.9±0.3	1.0 ± 0.2	0.7
DFA α2	1.1 ± 0.3	2.2 ± 5.9	0.5
***Brachial diameter variables***			
Baseline diameter, mm	2.8 ± 1	3 ± 0.7	0.43
Hyperemia diameter, mm	3 ± 0.9	2.9 ± 0.7	0.7
Δ FMD, mm	0.07 ± 0.8	-0.2 ± 0.7	0.71
% FMD	10.1 ± 23.3	-2.5 ± 15.5*	0.04
***Variables of Flow***			
Velocity baseline, cm/s	0.21 ± 0.14	0.13 ± 0.08*	0.02
Hyperemia flow velocity, cm/s	0.31 ± 0.12	0.2 ± 0.1*	0.001
Δ flow velocity, cm/s	0.02 ± 0.3	0.06 ± 0.2	0.4

Data in Mean ± SD. HR = heart rate; CO = cardiac output; SV = stroke volume; CI = cardiac index; SVR = systemic vascular resistance; RMSSD = square root of the difference in the sum of squares between R-R interval on the record, divided by the determined time minus one; SDNN = standard deviation of all average R-R intervals; Triangular index (RRtri) = histogram by density of normals RR intervals; LF = low frequency; HF = high frequency; LF/HF = sympathetic and parasympathetic component; SD1 = standard deviation of short-term HRV; SD2 = standard deviation measuring the dispersion of points along the line of identity; Student t test

*P<0.05.

**Table 3:** Predictive multivariate regression model of mortality, age and outcomes in studied patients.

Variables n = 60	Coefficient	Standard Error	*P values*
R^2^ = 0.72			
Constant	40.22	2.61	<0.001
Hypertension	19.26	2.94	<0.001
Diabetes Mellitus (DM)	3.31	3.58	0.35
Mortality	-3.07	2.87	0.29
FMD	-0.05	0.08	1.1

Age = 40.229 + (19.260 * Hypertension) + (3.318 * DM)—(3.074 * Mortality)—(0.0580 * FMD). Standard Error of Estimate = 10.340.

**Fig 3:**
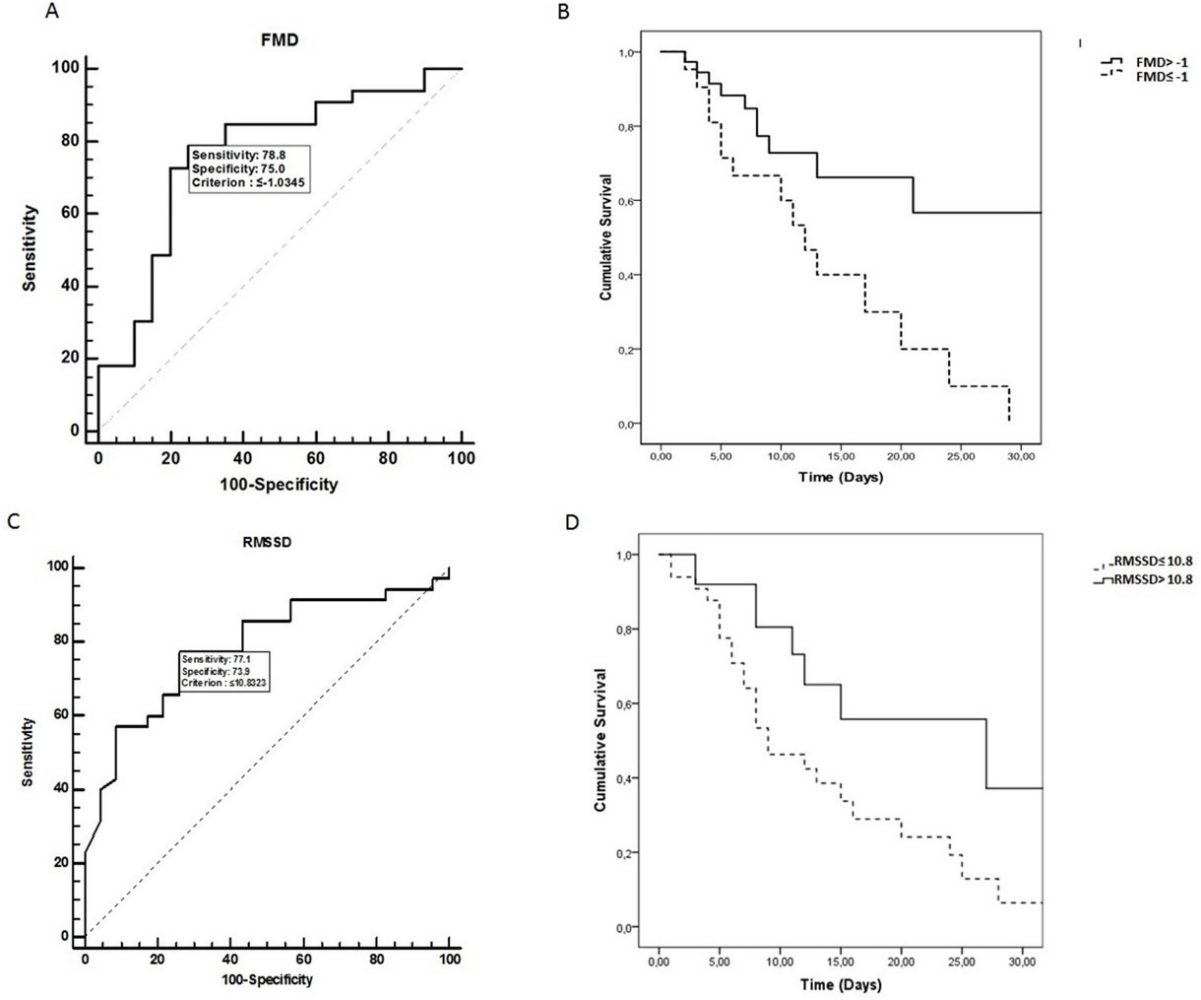
ROC and Kaplan-Meier curves for 28-day mortality in septic patients. A: The ROC Curve of FMD in predicting 28-day mortality in patients with sepsis. The area under the curve was 0.764 (0.627–0.869). The value of -1% was chosen as the cutoff point for FMD (sensibility of 78.7%, specificity of 75%, positive likelihood ratio of 3.15 and negative likelihood ratio of 0.28). B: Kaplan-Meier curve showing 28-day mortality in septic patients with FMD≤ -1% (mean survival time of 13.4 days) and FMD> -1% (mean survival time of 25.4 days). The survival curves were compared using log-rank test, p = 0.001. C: The ROC Curve of RMSSD in predicting 28-day mortality in patients with sepsis. The area under the curve was 0.784 (0.656–0.881). The value of 10.8 ms was chosen as the cutoff point for RMSSD (sensibility of 77.1%, specificity of 73.9%, positive likelihood ratio of 2.96 and negative likelihood ratio of 0.31. D: RMSSD≤10.8ms (mean survival time of 13.2 days) and RMSSD> 10.8ms (mean survival time 23.1 days). The survival curves were compared using log-rank test, p> 0.01, showing higher mortality in both lower cut-off.

Fig 3 illustrates the Kaplan-Meier curve showing 28-day mortality in septic patients with: 1) FMD≤-1% (mean survival time of 13.4 days and 95% Confidence Interval of 9.2–17.6 days) and FMD>-1% (mean survival time of 25.4 days and 95% Confidence Interval of 19.6–31.3 days); and 2) RMSSD ≤ 10.8ms (mean survival time of 13.2 days and 95% Confidence Interval of 9.6–16.8 days) and 2) RMSSD >10.8ms (mean survival time 23.1 days 95% Confidence Interval of 15.8–30.3 days). The survival curves were compared using a log-rank test demonstrating both were statistically significant (0.001 and 0.01, respectively), showing higher mortality for both lower cut-points.

## Discussion

In this prospective study, we characterized associated comorbidities, length of hospital stay and death rate of patients diagnosed with sepsis in a tertiary universitary hospital. In addition, we aimed to verify whether non-invasive methods of investigation, such as HRV indices, hemodynamic monitoring using cardioimpedance and endothelial function analysis, all noninvasive tools, could be predictors of length of hospitalization, ICU time, and short-term mortality in patients with sepsis.

The main findings can be summarized as follows: 1) we observed that high rates of ICU hospitalization and mortality; 2) we observed that patients who survived had greater responses to FMD% index, higher values of SD1 and triangular RR indices, and higher complexity indices, represented by SD1 when compared to patients who died. In addition, HR was lower in the surviving group; 3) it was observed that the RMSSD and SD1 indices may be predictors of endothelial function, represented by FMD indices (% FMD, FMD delta and FMD peak; and 4) SOFA, RMSSD and FMD can predict risk of death in these patients. These findings may be particularly relevant as they bring important information to clinical practice through noninvasive biomedical engineering methods, which can be easily obtained at the bedside for decision making in septic patients.

### Clinical characteristics of the septic patients

Our patients were selected from a population with a diagnosis of sepsis within the criteria established in the present study and who entered the ICU of a public hospital that serves a region of approximately 400,000 inhabitants. Many of these patients went through screening at other services such as basic health units and emergency services, remaining in emergency rooms or temporary units until they were referred to the ICU in question. This was promoted to frequently reach the ICU in a high degree of severity, observed in the high severity scores such as Apache and the highest mortality rate, when compared to the mortality found in the literature in sepsis [20]. In the present study, 30 cases of sepsis (50% of total) presented pulmonary focus infection, followed by 27 cases of abdominal focus infection, differening from the epidemiology reported in the literature [21], where the most frequent sepsis focus are pulmonary followed by urinary. It was observed that patients referred to the ICU were from several origins, from the surgical center in the postoperative cases, such as the emergency rooms of the hospital, and the stabilization rooms of the emergency medical service. This difficulty in the rapid mobilization of sepsis patients to the ICU can be an important factor for the high values in the severity scores, with a high percentage of estimated mortality, ~53% of predicted mortality by the APACHE score and the creatinine index around 2.4 mg/dL, which favored the elevation of the severity index as an important predictor of severity. The low pO_2_/FiO_2_ ratio and high values of blood lactate and leukocytes may have an important contribution to the reduction of the O_2_ extraction rate and for the presence of high SOFA. Thus, this value can demonstrate the severity of the patients at the time of admission to the ICU, since they were studied within the first 24 hours after sepsis diagnosis to our unit. Certainly, these data contributed to a high mortality rate found in this group of 65%, well above the high rate reported in 2017 in our country [21]. In addition, a period of hospital stay in the ICU of 10.4 ±8.2 days was evidenced, contrary to what is recommended by the sepsis Consensus [13].

### Clinical comparisons between survivor and non-survivor groups

In the present study, curiously the prevalence of death sepsis was significantly higher in women than in men. Although the mortality rate between genders is still a major challenge in sepsis, the results of the studies can be explained by the mechanism that determine gender dimorphism in the immune system. Thus, the improvement of immune function in women may be related to the high prevalence of autoimmune diseases, which can be caused by the absence of androgenic hormones and immunosuppressants [22]. According to Schroder et al, the reduction in the function of macrophages and lymphocytes after a hemorrhagic shock has been more evident in men when compared to women because of female immunity. In this sense, it can be suggested that low levels of testosterone or increased levels of estradiol can produce a better prognosis in women with sepsis [22]. However, since these hormonal levels were not measured in the present study, it is suggested that the age factor (55±11 years) found for NSG was relatively higher when compared to the survivors group, to the hormonal reduction with consequent change in the immune mechanism of this group, and increased mortality. Another factor to be analyzed is the fact that the group studied was not selected according to gender and it is not a question of two homogenous groups of only different genders, with similar infections and clinical conditions, where it would be possible to compare them in the results. For this reason, future studies are needed to assess the pathophysiology and prognosis in the context of sex-specific hormones.

On the other hand, as expected, APACHE values, APACHE mortality (%), SOFA score and the focus of pulmonary sepsis were higher for the NSG compared to the SG (P<0.05). Similar results were observed by Chen closet al. in 2011, where higher APACHE values and SOFA score with higher mortality [23]. Furthermore, higher amounts of drugs (noradrenaline and sedative) were administered in the NSG in contrast to the SG, demonstrating advanced severity in clinical status; these patients had prolonged time inintensive care relating to the highest APACHE indices. It is known that the time of hospital stay and hemodynamic instability may provide increased administration of sedatives and vasoactive drugs with the goal of balancing hemodynamics and maintaining macrocirculation [24].

### Hemodynamic, autonomic and endothelial function alteration between surviving and non-surviving sepsis groups

Hemodynamic changes are common manifestation during sepsis. In the present study, we observed that NSG presented higher values of HR in comparison to the SG, measured by noninvasive transthoracic cardioimpedance. The hypotension present in sepsis is secondary to the reduction of systemic vascular resistance with reduced pre and afterload, unstable filling conditions, diastolic and LV systolic dysfunction, catecholamine elevation and chronotropic dysregulation [25]. In addition, myocardial contractility is compromised early in sepsis which was previously confirmed by echocardiography [26]. Tachycardia is commonly observed in patients with sepsis, explained by adrenergic stimulation and fever [27]. However, in the present study our patients were in controlled temperature and hemodynamic stability. A decrease in the response to catecholamines by myocardial receptors, in addition to high HR can further potentiate myocardial compromise, leading to arrhythmias and impairment of diastolic function and filling [25].

We observed that the indices representative of vagal modulation and total HRV (Triangular Index and SD1) were reduced in patients who subsequently died. Therefore, we can infer that such indices, when evaluated at an early stage, may indicate early autonomic dysfunction and consequently the death of these patients. Studies have suggested that changes in hemodynamic and cardiac autonomic function may be risk factors for adverse events; however, other studies have also reported increased cardiac involvement in surviving patients compared to non-survivors of sepsis. [28]. The mechanisms of cardiac dysfunction in sepsis have been extensively reviewed by other investigators [29], in addition to other markers such as high levels of autonomic system catecholamines [30]. An important mechanism of sepsis-induced cardiac dysfunction is the attenuation of adrenergic response at the cardiomyocyte level due to low regulation of beta-adrenergic receptors and post-receptor signaling pathway depression [31]. These changes are mediated by cytokines and nitric oxide [32]. Finally, the optimization of the preload with the tachycardia resulting from the actions of the catecholamines can generate elevation of CO, in spite of deep myocardial depression.

Regarding the results of the present study on endothelial function, evaluated noninvasively by FMD, we observed that the surviving patients presented higher values of % of FMD, velocity baseline, and hyperemia flow velocity when compared with the NSG. This finding suggests a deterioration of the vascular function associated with the inflammatory process and the severity of the disease in patients with sepsis. Additionally, organic dysfunction in sepsis may be cumulative on mortality, in which it may reach high rates in cases of sepsis with severe hypotension [33]. The endothelium may play an important role, producing biological and vasomotor mediators in order to balance the release of nitric oxide and endothelin into the bloodstream [34].

In a study conducted by Becker in 2012, where they evaluated FMD in patients with sepsis and septic shock and compared with controls at baseline, 24hs and 72hs after ICU admission, they showed a decline in FMD (5.2±4 for survivors and -3.3±10 for non-survivors), especially 72h after admission. The authors concluded that FMD changes occur early in septic patients with hemodynamic instability, with progressive FMD reduction in the first 72 hours, thus representing a worse prognosis [10]. In our study, we also observed a marked reduction of FMD in septic patients, especially in NSG, however, our mean values were much lower than those measured in Becker's study [10]. This finding again suggests that a deterioration in vascular function assessed by FMD is entirely associated with the severity of sepsis.

### Correlations, simple linear regression and multivariate regression model

In the present study, interestingly, we observed that RMSSD and SD1 indices, both representative of vagal modulation, correlated positively and moderately with parameters of endothelial function, represented by vasodilatation mediated by brachial artery flow. It is widely recognized that endothelial function is considered to be an independent predictor of risk of future cardiovascular events in patients with cardiovascular disease and non-cardiovascular disease [35], as well as a strong predictor for complications in the postoperative period of cardiovascular surgery. In addition, it is known that HRV indices are also strong independent predictors of cardiovascular risk, cardiovascular and non-cardiovascular death and complications [36]. In this sense, we compared the HRV and FMD indices and found that the RMSSD and SD1 indices were predictors of % FMD, Delta FMD and FMD peak. Considering the costs involved in the techniques, HRV can be a low-cost and easily obtainable method, since it only requires previous training for index calculations and can be easily obtained, while the FMD requires more training and high cost of equipment used. Although expected, it was observed in the present study, influence of several clinical variables for prognostic definition and mortality in sepsis. The data in Table 3 demonstrate that the variables that presented statistical values for determining the predictive model for mortality were age and hypertension. A recent study by Kim et al. in 2019 showed the results were worse in patients who did not receive medications for hypertension in the period of 1 month before the diagnosis of sepsis, evidencing increasing hospital stay rate and higher mortality when compared to non-medication patients [37].

### Survival and risk indices in sepsis patients

Interestingly, in the present study, we found that Kaplan-Meier survival curve analysis revealed that RMSSD above and below a 10.8ms cut-off discriminated between survivors and nonsurvivors. In addition, patients who had FMD% values above -1 presented with greater survival and a consequent reduction in risk of death at 28 days. Although there is no previous study demonstrating the cut-off values of RMSSD indice for septic patients, de Castillo closet al. reported on a cut-off of ≤17ms for the SDNN indexthat expressed total variability. In this context, to our knowledge, our study is the first to contrast survivors and nonsurvivors of sepsis, using the parassimpathetic index of HRV. Moreover, previous studies showed that RMSSD may be a novel and independent risk factor for mortality in other clinical conditions [9].

Interestingly, it was also observed in our study that patients who presented with a baseline FMD% cut-off score above -1 had a longer survival and lower risk of death at 28 days. To date, no study has shown such a finding, however, it may be related to FMD and the presence of nitric oxide, which in turn is known to represent an important predictor of organ dysfunction, as well as a marker of mortality in patients with sepsis and septic shock [38,39]. Thus, nitric oxide dysregulation is a probable cause of vascular insensitivity to stress in sepsis [6].

However, it is clear that FMD does not depend exclusively on nitric oxide, but on several other endothelium-dependent mechanisms, such as prostaglandins and the hyperpolarizing endothelial-derived factor. In the present study, the reduction of FMD was associated with the risk of death for sepsis. Thus, our findings may help guide clinicians to implement appropriate treatment strategies in sepsis, implementing more aggressive intervention strategies when appropriate. Thus, our findings may guide clinicians to implement this important tool to detect patients with higher risks.

### Study limitation

The present study presents with some limitations that should be considered. The main limitation is the small number of patients with sepsis. Another important factor is related to the capture of signals in the ICU, since it was necessary to exclude some signals of HR by telemetry and cardioimpedance. Moreover, other possible influences may have influenced HRV analysis, such as the use of sedatives, vasoactive drugs and the use of mechanical ventilation; however, the presence of these conditions are intrinsically related to patients at risk for sepsis making our findings applicable in a real-world clinical setting. Furthermore, no adjustments were made for confounding factors, especially for severity scores as SOFA, APACHE II and medication (vasoactives drugs that may deeply influence in mortality, and hospitalization status. In addition, the present study controlled for other influences that could influence data collection such as, such as collecting data points the same time of day to avoid circadian interferences.

## Conclusions

We can conclude, based on the findings of the present study, that noninvasive measurements of cardioimpedance, HRV and FMD in septic patients can indentify survivors and nonsurvivors. These findings may indicate new markers to assess the risk of death in sepsis. Such results can add relevant information in clinical practice, since such noninvasive methods are easily obtained at the bedside in septic patients, being promising tools of applied biomedical engineering in the context of health-disease.

## Supporting information

S1 Table Complete list of characteristics, clinical and mortality data. (DOCX)
