# Correction: Noninvasive measurements of hemodynamic, autonomic and endothelial function as predictors of mortality in sepsis: A prospective cohort study

**DOI:** 10.1371/journal.pone.0216505

**Published:** 2019-04-30

**Authors:** The PLOS ONE Staff

## Notice of Republication

An incorrect version of Fig 2 was published in error. The publisher apologizes for this error. This article was republished on April 23, 2019 to correct for this error. Please download this article again to view the correct version.

As a part of the republication, the affilation for the Corresponding Author, Audrey Borghi-Silva, was also corrected. In the originally published article, the Corresponding Author was incorrectly affiliated with #2. The correct affiliation is: #3 Cardiopulmonary Physical Therapy Laboratory, Federal University of Sao Carlos, Sao Carlos, Brazil.
